# Hospitalizations of Patients with Atrial Fibrillation (AF) in Poland: A Nationwide Study Based on over One Million AF Hospitalizations in 2017–2021

**DOI:** 10.3390/jcm13216592

**Published:** 2024-11-02

**Authors:** Martyna Dąbrowska, Michał Rząd, Krzysztof Kanecki, Katarzyna Lewtak, Piotr Tyszko, Paweł Goryński, Aneta Nitsch-Osuch

**Affiliations:** 1 Department of Social Medicine and Public Health, Medical University of Warsaw, 02-106 Warsaw, Poland; 2 Department of Cardiology and Internal Medicine, Military Institute of Medicine—National Research Institute, 04-141 Warsaw, Poland; 3 Institute of Rural Health in Lublin, 20-090 Lublin, Poland; 4 National Institute of Public Health NIH—National Research Institute, 00-791 Warsaw, Poland

**Keywords:** atrial fibrillation, epidemiology, hospitalization rates, population study, epidemiological trends, in-hospital mortality

## Abstract

**Background/Objectives:** Atrial fibrillation (AF) is one of the most common forms of persistent arrhythmia in adults worldwide. The aim of this study was to present recent data on the epidemiology of patients hospitalized with AF in Poland. **Methods:** This is a retrospective, population-based study conducted using hospital discharge records. The data covered 1,225,424 cases of AF hospitalization reported in 2017–2021. **Results:** The study group consisted of 51.36% men and 48.64% women. The mean and median ages were 73.6 and 74 years, respectively. Women were older than men (77 vs. 70 years, *p* < 0.001). The mean and median lengths of hospitalization were 6.9 and 4 days. The mean annual hospitalization rate was 640.0 per 100,000 person-years. In the group of patients aged ≥65 years, the hospitalization rate was 2870.4 per 100,000 person-years. Men were hospitalized more frequently than women (*p* < 0.001). The total in-hospital mortality rate was 37.7 per 100,000 person-years, and it was higher in women than in men (*p* < 0.001). There was a significant downward trend in first-time hospitalizations during the analyzed period and a significant downward trend in mortality rates with a marked increase in the years 2020–2021. **Conclusions:** Although women are less frequently hospitalized for AF, they show a higher risk of fatal hospitalizations. The pandemic may have reduced new AF diagnoses and increased mortality in this group of patients. The results of this study may be helpful in making comparative analyses in the European and global contexts and taking actions aimed at improving the health condition of the Polish population.

## 1. Introduction

Atrial fibrillation (AF) is one of the most common forms of arrhythmia in adults worldwide [1,2]. AF is a supraventricular tachyarrhythmia, leading to uncoordinated electric activity of the atrium and, consequently, to ineffective atrial contraction. Increasing age is the most important risk factor for atrial fibrillation [3]. Among other known, unmodifiable risk factors are genetic factors, male sex, and Caucasian ethnicity [3,4,5]. The increasing burden of comorbidities such as arterial hypertension, diabetes mellitus, heart failure (HF), coronary artery disease (CAD), chronic kidney disease (CKD), obesity, and obstructive sleep apnea (OSA) is also important in the pathogenesis and development of AF [6,7,8,9]. The lifetime risk of developing AF is estimated at about one in three individuals of European ancestry and one in five in African American individuals [10].

Patients with AF can present with a range of symptoms, from palpitations, dyspnea, and poor exercise tolerance to syncope and hemodynamic instability, although many of them are asymptomatic [11,12]. AF is associated with an increased risk of ischemic stroke or systemic embolism. The neurological consequences of AF may significantly negatively impact patient survival, constitute a significant burden for the patients and their families, and be a significant cost factor for the health care system [3,13]. It is also a risk factor for other conditions such as HF, reduced exercise capacity, reduced quality of life, the development of a disability, mood disorders, and cognitive impairment, including dementia [13,14,15].

Current knowledge of AF incidence, prevalence, risk factors, and associated cardiovascular morbidity and mortality is derived from long-running cohort studies, mainly in North America, or more recently, from registries based on the International Classification of Diseases (ICD) discharge codes. The 2019 Global Burden of Disease study provides the latest AF and atrial flutter (AFL) data. In 2019, AF/AFL affected about 60 million people worldwide. The number of incident AF/AFL cases in 2019 was 4.7 million, and the number of AF/AFL-related deaths worldwide was 0.32 million. Considering sex, there were more AF/AFL cases in men than women in 2019 [16]. According to the Framingham Heart Study, the incidence of AF has increased by over 3 times over the last 50 years [17]. In a Dutch study from 2015, the overall incidence rate of AF was 3.3 per 1000 person-years, and it was higher in men than in women, with a strong increase with age [3]. According to a study of an 8.3 million population from Germany, the AF prevalence was 2.132%, and the incidence of AF in that sample was 4.358 cases per 1000 person-years in men and 3.868 cases per 1000 person-years in women [18]. According to a large populational study from England, the standardized AF incidence increased by 30% over 20 years of observation. Due to the increasing age of the population, the absolute number of incident AF cases increased by 72%. The age-standardized incidence was higher in men than in women, and men were younger at diagnosis [19]. In Europe, the prevalence of AF in 2010 was estimated at ~9 million individuals older than 55 years, and by 2060, it is expected to reach 14 million [20,21]. In the United States (US), at least 3–6 million persons suffer from AF, and these numbers are predicted to reach 6 to 16 million by 2050 [22,23]. Based on a recent study from Poland, the prevalence of AF in the Polish population aged over 65 years was estimated at 19.2%. [24]. Atrial fibrillation is associated with an increased risk of at least one hospitalization per year, which, given its increasing prevalence, represents a severe financial burden for health care systems [25,26]. In Denmark, AF (primary or secondary diagnosis) accounted for 6.8% of hospital admissions in a 13-year period of observation (1997–2009) [27].

The analyzed period is particularly specific because it also includes two years of the COVID-19 pandemic, which began on 11th March 2020. The pandemic has contributed to vital changes in the organization of health care systems worldwide, prioritizing COVID-19-related hospitalizations and deferring most elective hospital admissions. It has become a burden to public health, resulting in a decrease in hospital admissions of patients with chronic diseases [28].

The aim of this study was to present recent data on the epidemiology of hospitalizations of patients with AF in Poland in the years 2017–2021.

## 2. Materials and Methods

### 2.1. Patients and Methods

This study is a retrospective, population-based study conducted on hospital discharge records that included a diagnosis of AF. Data were obtained from the National Institute of Public Health in Poland and covered 1,225,424 cases of AF hospitalizations reported in the years 2017–2021. All hospitals in Poland, except for psychiatric facilities, are legally required to submit discharge data to the National Institute of Public Health. The data are anonymous and contain information on diagnosis codes according to the International Statistical Classification of Diseases and Related Health Problems, Tenth Revision (ICD 10), hospital admission and discharge data, and the sex, date of birth, and place of residence of the patient. Patients included in the study were hospitalized with ICD 10 diagnosis code “I48” (primary or secondary diagnosis), which is attributable to the diagnosis of AF. The authors assumed that in all cases, AF was diagnosed according to the most current and widely used criteria, which means that diagnosis was confirmed by characteristic abnormalities in an ECG recording. In this article, we used the term “first-time hospitalization” to mean the patients’ first hospitalization in the period analyzed. Subsequent hospitalizations in the analyzed period were identified by comparing date of birth, sex, and residence code. Since all hospitalizations in Poland (except psychiatric ones) are registered and reported for epidemiological surveillance, informed consent or approval by a medical ethics board was not required under national regulations.

### 2.2. Statistical Analysis

To perform most statistical analyses, Statistica (TIBCO Software Inc., version 13) [29] was used. WINPEPI [30] was used to perform chi-square tests. For continuous variables with a normal or non-normal distribution, respectively, means and 95% confidence intervals or medians and IQRs were computed. For nominal variables, counts and percentages were analyzed. Rates of hospitalizations and in-hospital mortality related to AF were calculated as the estimated number of hospitalizations or deaths per 100,000 person-years using data (national census) from Statistics Poland [31]. When comparing the two groups in terms of hospitalization or mortality rates, the odds ratio (OR) was reported. To assess trends, linear regression was applied. When reporting linear models, the coefficient of determination (R^2^) was presented. To assess normal distribution, the Shapiro–Wilk test was used. When normality assumptions were not met, non-parametric tests (chi-square, U Mann–Whitney) were applied. A two-sided *p*-value less than 0.05 was considered statistically significant.

## 3. Results

**Figure 1:**
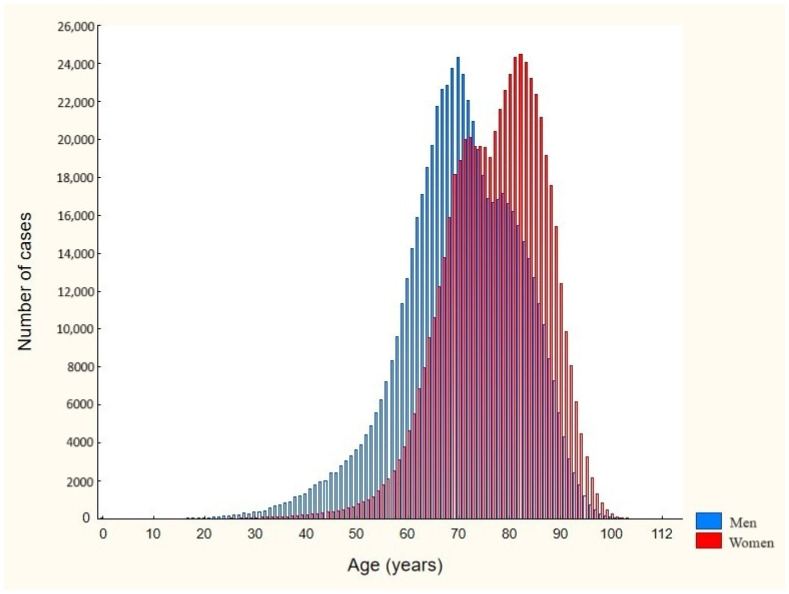
Age distribution by sex among hospitalized patients with AF in Poland, 2017–2021.

During the study period, a total of 1,225,424 hospitalizations of AF patients were recorded. The study group consisted of 629,324 men (51.36% of all patients) and 596,086 women (48.64% of all patients), and in 14 cases, sex was specified as neither male nor female. In 32.4% of all hospitalizations, AF was the primary diagnosis. The mean and median ages in the presented population were 73.6 (95% CI: 73.6–73.6) and 74 (IQR 67–82) years, respectively. As shown in Figure 1, the hospitalized women were significantly older than the hospitalized men (mean age 77 vs. 70 years, *p* < 0.001). The mean and median durations of hospitalization were 6.9 (95% CI: 6.8–6.9) and 4 (IQR 2–8) days, respectively.

**Figure 2:**
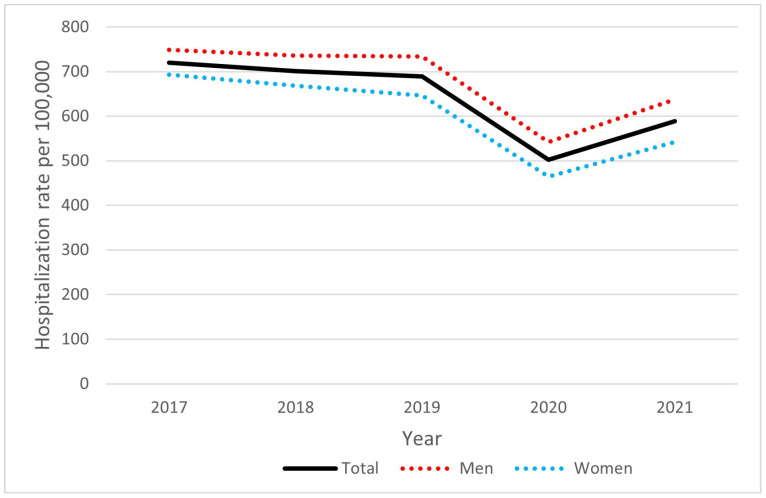
AF hospitalization rate in Poland by sex, 2017–2021.

As shown in Figure 2, the overall hospitalization rate was 640.0 per 100,000 person-years, and men were hospitalized more frequently than women (679.9 per 100,000 person-years and 603.1 per 100,000 person-years, respectively, OR 1.13, *p* < 0.001). In the group of patients aged 65 years and over, the hospitalization rate was 2870.4 per 100,000 person-years, and it was higher among men than women (3304.2 and 2583.8 per 100,000 person-years, respectively, OR 1.28, *p* < 0.001). No significant fluctuations were observed for the annual number of hospitalizations in subsequent years, but a decrease in hospitalizations for atrial fibrillation was noted in 2020 and 2021. A seasonal pattern was not observed.

**Figure 3:**
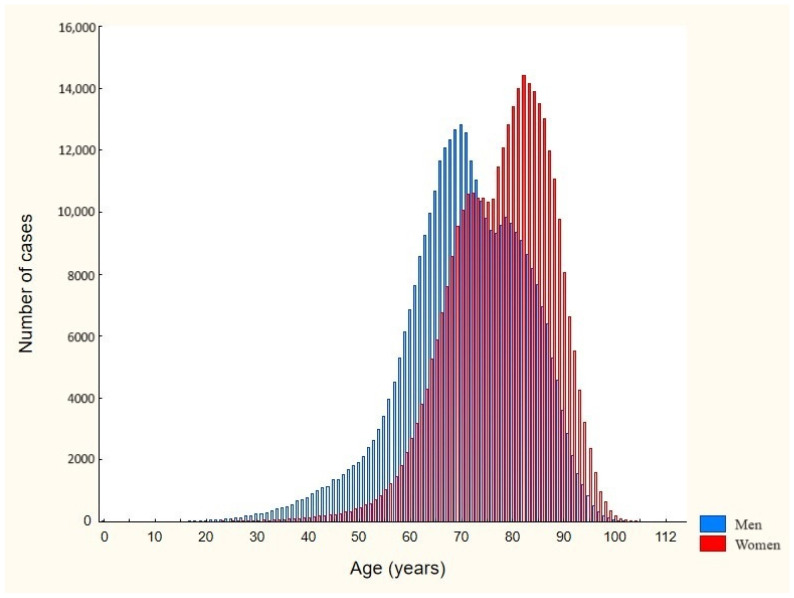
Age distribution by sex at first-time AF hospitalization in Poland during the study period, 2017–2021.

**Figure 4:**
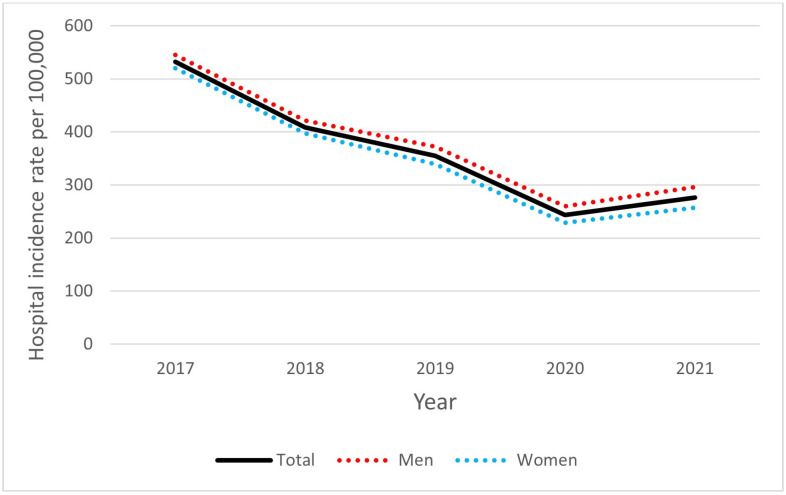
AF first-time hospitalization rate in Poland by sex, 2017–2021.

In the study period, 56.8% (*n* = 695,618) of all hospitalizations were first-time admissions. The average number of hospitalizations was 1.76 per patient. The mean and median ages were 74.1 (95% CI: 74.1–74.2) and 75 (IQR 67–83) years, respectively. As shown in Figure 3, women were significantly older than men (mean age 78 versus 71 years, *p* < 0.00001). For this subgroup, the hospital incidence rate was 363.6 per 100,000 person-years, and it was higher in men than in women (379.5 and 348.8 per 100,000 person-years, respectively, OR 1.09, *p* < 0.001). As visualized in Figure 4, there was a significant downward trend in first-time hospitalizations during the analyzed period (R^2^ = 0.87, *p* < 0.05). In the group of patients aged 65 years and over, the hospital incidence rate was 1640.6 per 100,000 person-years, and it was higher among men relative to women (1858.8 and 1496.3 per 100,000 person-years, respectively, OR 1.24, *p* < 0.001).

**Figure 5:**
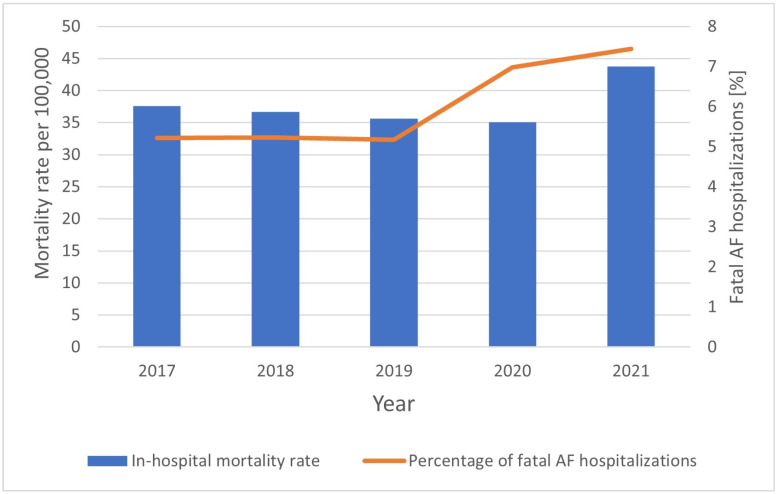
Mortality by year during AF hospitalization in Poland, 2017–2021.

Among all cases of hospitalization that took place during the study period, 72,191 in-hospital deaths were reported (5.9%). In-hospital mortality was higher in women than in men (39,611 (11.5% of uniquely identified patients) and 32,580 (9.3%), respectively, *p* < 0.001). Regarding demographic data, the total in-hospital mortality rate in patients with AF was 37.7 per 100,000 person-years, and it was higher in women than in men (40.1 and 35.2 per 100,000 person-years, respectively, OR 1.14, *p* < 0.001). An analysis of the graph (Figure 5) showing the change in the in-hospital mortality rate over the study period revealed a significant downward trend in the years 2017–2020 (R^2^ = 0.99, *p* < 0.01), and a marked increase in 2021. Furthermore, an analysis of the percentage of fatal hospitalizations due to AF shows that after a relatively stable period in 2017–2019, a significant increase in this value can be observed in 2020–2021.

## 4. Discussion

During a 5-year period from 2017 to 2021, over a million hospitalizations with a diagnosis of AF were reported in Poland. Men accounted for more than a half of the reported hospitalizations, and they were younger than the hospitalized women. However, women were observed to have higher in-hospital mortality rates.

In our study, the overall hospitalization rate was 640.0 per 100,000 person-years. We observed a non-significant decline in the number of AF hospitalizations in the pandemic years. According to statistics from the US, the AF hospitalization rate in 2010 was 1812 out of a population of 1 million, and this was an increase of 14.4% compared to the year 2000. Throughout the study period, the AF hospitalization rate was higher in women and persons of Caucasian origin [32]. In a study by Naderi et al. [33], hospitalization rates increased with age, and they were the highest in the group of persons aged 85 years and over, in which the incidence accounted for 1367 per 100,000 person-years.

The proportion of men in the study group was 51.36%. Comparable findings were reported in other studies on AF hospitalizations carried out in Denmark, France and Germany, with estimates of 53.18%, 57.7% and 53.7%, respectively [27,34,35]. The increased incidence of AF among men that was observed in other studies may be due to an elevated prevalence of risk factors in that sex.

The mean and median ages of the hospitalized patients in Poland were 73.6 and 74 years, respectively. In the previously mentioned American register, the mean age of patients hospitalized with a primary diagnosis of AF was 70 years, 66 years for men and 74 years for women [32]. In another study that was also conducted on the American population in the years 2009–2010, the mean age of hospitalized patients was 70 years, but 67% of the participants were over 65 [33]. Danish findings showed that the median age of hospitalized patients with a diagnosis of AF was 75.65 ± 12.76 years [27]. Compared to the Polish population, patients from both studies from the US were younger. This may result from the fact that the abovementioned studies concerned hospitalizations for AF as a principal diagnosis, whereas our database concerned hospitalizations for AF as a primary or secondary diagnosis.

Hospitalized women were older than hospitalized men (mean age 77 vs. 70 years). Similar results were found in an American publication, with women being significantly older than men (mean age 74 vs. 67 years) [36]. In data on a Chinese population of hospitalized AF patients, females were significantly older than men (mean age 71.8 ± 10.4 vs. 69.6 ± 11.8 years), and there were more females aged over 75 years [37]. In a study by Lip et al. on inpatients and outpatients with AF presenting to cardiologists, the female participants were found to be older, with a higher proportion of those aged over 75 years old [38].

The mean and median durations of hospitalization were 6.9 and 4 days, respectively. In an article by Naderi et al. [33], the average length of hospital stay was 3 days, although more extended hospital stays were observed in patients over 65 years of age: 3.7 days versus 2.9 days. When compared with our results, the length of hospital stay was shorter among the patients from the USA. Nevertheless, the sample on which that study was conducted included patients discharged from acute-care, short-term hospitals, with AF as a principal diagnosis. Results that were similar to ours were reported in a Danish publication, where the mean length of hospital stay in patients with AF was 5.5 days (SD 8.7). The database included all hospitalizations with a diagnosis of AF. In addition, patients with AF were hospitalized longer for any reason (cardiovascular and non-cardiovascular) than patients without AF [27]. According to the Chinese registry, the median length of hospital stay was 10 days (IQR 7–14 days) [37]. The prolonged hospitalization of patients with AF is associated with a higher economic burden for the entire health care system.

In the study period, 56.8% of all hospitalizations were identified as first-time hospitalizations. This translated into an average number of hospitalizations of 1.76 per patient. A study from Denmark raised a similar issue, as it indicated that 57.4% of patients were readmitted to hospital during a follow-up period of 6 years [27]. The phenomenon of re-hospitalizations results in an additional burden on the health care system and requires special consideration in order to reduce the need for hospital intervention and thus reduce the cost of care for patients with AF.

In the present study, the mortality rate was 5.9%. A French register showed that patients hospitalized with AF as the principal diagnosis presented an attributable mortality of 0.6%, and the mortality in patients hospitalized with AF associated with another pathology revealed a rate of 6.6%, which resulted in an all-cause hospital mortality of 5.6% [34]. That is in line with our results. However, different data are observed in studies from the USA, where in-hospital mortality was estimated at 0.9% [32]. In another study, the total mortality rate was calculated as 0.8%. Mortality in patients aged 65 and over reached 1%. [33] Nevertheless, the sample on which the study was conducted included only patients discharged from the hospital with AF as a principal diagnosis. Our study included hospitalized patients with AF as a primary or secondary diagnosis, which is why the mortality rate may be higher. A recent study from Thessaloniki estimates mortality from any cause in AF patients after a median follow-up of 31 months after hospitalization at 37.3% [39].

The mortality rate was reported to be higher in women than in men (40.1 and 35.2 per 100,000 person-years, respectively). An increased standardized mortality ratio was also reported in women with AF in a 2018 Korean study, which showed results of 3.81 in women versus 3.35 in men [40]. These results are consistent with data from a Swedish register, where the relative risk for all-cause mortality after adjustment for age and comorbidities was higher in women than in men in all age groups participating in the study in the first year after AF diagnosis [41]. Similarly, in a US study based on the National Inpatient Sample of 82,592 patients hospitalized in 2018 with a primary diagnosis of AF, women had a relatively higher in-hospital mortality (0.9% vs. 0.8%, *p* = 0.070), but after adjustment for known risk factors, female sex was no longer a significant predictor of mortality [36]. This may be because women tend to be older and more symptomatic and have a poorer health status than men [38]. In addition, women tend to be diagnosed with AF at an older age, which is associated with a greater burden of comorbidities [41].

In our study, we did not observe significant fluctuations in the annual number of hospitalizations in subsequent years; however, there was a decrease in the number of hospitalizations for atrial fibrillation in 2020 and 2021. Moreover, the number of hospitalizations in 2021 did not reach the pre-pandemic level. An analysis of first-time hospitalizations over the study period showed a significant downward trend, with the lowest number in 2020–2021. This may result from the fact that most elective hospitalizations were postponed or rescheduled during the national lockdown, when access to health care providers was limited and many visits had to be conducted remotely, without a physical examination or electrocardiogram. Moreover, patients were advised to avoid social interactions and unnecessary visits to medical facilities, which means that those with mild to moderate symptoms of AF were probably not referred to emergency departments (EDs) or outpatient clinics. Another factor that may have influenced the trends in hospitalizations is the use of a more comprehensive approach to patients with AF using the ABC pathway. The ABC pathway is a holistic approach suggested by the European Society of Cardiology, and the acronym means A—avoid stroke with anticoagulation, B—better symptom control, and C—cardiovascular risk factor and comorbidity management. Adherence to the ABC pathway was associated with a reduced risk of adverse outcomes in a large study on an Asian population [42]. In another study, patients who received integrated care supported by telemedicine had a lower risk of re-hospitalization [43]. As promising as these results are, the use of telemedicine to reduce hospitalizations is still not as widespread in Poland. When analyzing the reasons for the reduction in hospitalizations, the CASTLE-AF trial is worth mentioning, in which patients with heart failure with a reduced ejection fraction and AF who underwent catheter ablation had significantly lower rates of death and hospitalization [44]. A decrease in AF hospitalizations was also observed in a study from a large center in Poland; however, this study compared the years 2019 and 2020. In 2020, there was a decline in AF hospitalizations by 59.72% compared to 2019, which resulted from the national lockdown. After the lockdown, the number of AF hospitalizations rose steadily, but it never reached the pre-pandemic levels [45]. In our study, we did not observe such a decrease; the total number of AF hospitalizations in 2020 decreased by 27.4% as compared to 2019, but it was still not significant. The report on the health status of the Polish population, prepared by the National Institute of Public Health NIH—National Research Institute—included aggregate causes of hospitalizations in 2019–2021. The authors of the report indicated a decrease in the number of all hospitalizations, including cardiovascular hospitalizations, in 2020 as compared with the previous year and a subsequent increase in 2021, which, however, did not reach the pre-pandemic levels [46]. A similar trend regarding AF was observed in our study. In a German study performed during the lockdown in the year 2020, a decrease in AF hospitalizations was observed as compared to the previous year [35]. Similar trends were observed in a recent American study, where there was a significant decrease in AF-related hospitalizations during the first year of the COVID-19 pandemic outbreak [47]. In a Danish study comparing the number of new-onset AF (NOAF) cases during the first 3 weeks of national lockdown to the same period in the previous year, a 47% drop was reported, which is in line with our results. Patients diagnosed with NOAF were younger, with a lower CHA_2_DS_2_-VASc score, but with a higher prevalence of cancer, heart failure and vascular diseases [48].

We also observed a significant downward trend in the in-hospital mortality rate between the years 2017 and 2020 (R^2^ = 0.99, *p* < 0.01) and a marked increase in the rate in 2021, as shown in Figure 5. According to a study covering data from the Silesian voivodeship in Poland, AF patients admitted to hospital during the pandemic were older and had more comorbidities, such as arterial hypertension and coronary artery disease. They were also more likely to have suffered from myocardial infarction or ischemic stroke in their previous medical history [45]. In a study from Germany, AF patients hospitalized during the pandemic had congestive heart failure more frequently [35]. Figure 5 shows a marked increase in the percentage of fatal hospitalizations due to AF during the pandemic period. This may be due to both the impact of SARS-CoV-2 infection on the severity of symptoms originating from the cardiovascular system, which can destabilize this group of patients, and the accumulation of the so-called health debt, which resulted from the reorganization of health care during the pandemic, poorer access to medical services, patients’ fear of COVID-19 and thus their delay in visiting health care professionals [46]. Moreover, a significant increase in the composite endpoint of mortality/ACS/stroke was observed during the COVID-19 outbreak in a previously mentioned American study. The in-hospital mortality in 2020 was higher compared to the pre-COVID-19 era [47].

Our study has several limitations. It analyzed trends in patients hospitalized with an AF diagnosis without conducting broader comparative analyses, such as an analysis of the causes of death and comorbidities related to changes in epidemiological trends over time. The personal COVID-19 status of the patients was not verified. All these issues and other interesting topics will be the subject of separate analyses. During this study, verifying the correctness of the diagnosis and reporting of the disease was not possible, but it was assumed that physicians used the most current, commonly used criteria and that AF was confirmed by ECG registration. In addition, the national database, which was the original source of data, did not include information on the temporal trend of AF (paroxysmal/persistent, permanent). Furthermore, only hospital discharge records were included in the database, which means that some outpatients with AF were excluded from this study. However, the considerable length of the observation period and the large size of the data sample obtained from the National Institute of Public Health are of great benefit to this study. The data include hospitalizations from the entire country, not just from specific centers, which could distort the calculated hospitalization rates. In addition, this study includes more than 1,225,000 patients, which allows for accurately reflecting epidemiological trends among AF patients. Moreover, the data used are automatically reported in accordance with the law and are not subject to bias associated with active reporting by physicians. An additional advantage of this dataset is that it is closely linked to administrative and social reports, which allows for a direct comparison between national census data and the reported prevalence and refinement of the results obtained with regard to the number of inhabitants.

## 5. Conclusions

To the best of the authors’ knowledge, this study presents, for the first time, aspects of AF based on the national hospital morbidity registry and presents the latest data on AF epidemiology in Poland. Given the increasing prevalence of AF worldwide, aging populations, improved methods of diagnosis and the increasing burden of comorbidities (which are risk factors for the development of AF), AF remains a significant public health problem in Poland. Although women are hospitalized for atrial fibrillation less frequently, they are at an increased risk of fatal hospitalizations. The pandemic may have both reduced the number of new diagnoses of atrial fibrillation and increased mortality among patients with this type of tachyarrhythmia. The results of this study may be helpful in making comparative analyses in the European and global contexts and taking actions aimed at improving the health condition of the Polish population.

## Data Availability

Nationwide General Hospital Morbidity Study, National Institute of Public Health NIH-National Research Institute, Warsaw, Poland.
